# Environmental Pollution to Blame for Depressive Disorder?

**DOI:** 10.3390/ijerph19031737

**Published:** 2022-02-02

**Authors:** Mariana Segovia-Mendoza, Margarita Isabel Palacios-Arreola, Lenin Pavón, Luis Enrique Becerril, Karen Elizabeth Nava-Castro, Omar Amador-Muñoz, Jorge Morales-Montor

**Affiliations:** 1 Departamento de Farmacología, Facultad de Medicina, Universidad Nacional Autónoma de México, Ciudad de Mexico 04510, Mexico; mariana.segovia@facmed.unam.mx; 2 Grupo de Especiación Química de Aerosoles Orgánicos Atmosféricos, Instituto de Ciencias de la Atmósfera y Cambio Climático, Universidad Nacional Autónoma de México, Ciudad de Mexico 04510, Mexico; margarita.palacios@atmosfera.unam.mx (M.I.P.-A.); oam@atmosfera.unam.mx (O.A.-M.); 3 Laboratory of Psychoimmunology, National Institute of Psychiatry Ramón de la Fuente Muñiz, Calzada México-Xochimilco 101, Colonia San Lorenzo Huipulco, Tlalpan, Ciudad de Mexico 14370, Mexico; lkuriaki@imp.edu.mx (L.P.); lusenbeve@yahoo.com (L.E.B.); 4 Grupo de Biología y Química Atmosférica, Instituto de Ciencias de la Atmósfera y Cambio Climático, Universidad Nacional Autónoma de México, Ciudad de Mexico 04510, Mexico; karlenc@atmosfera.unam.mx; 5 Departamento de Inmunología, Instituto de Investigaciones Biomédicas, Universidad Nacional Autónoma de México, Ciudad de Mexico 04510, Mexico

**Keywords:** depression, serum levels, phthalates, bisphenols

## Abstract

Public concern has emerged about the effects of endocrine-disrupting compounds (EDCs) on neuropsychiatric disorders. Preclinical evidence suggests that exposure to EDCs is associated with the development of major depressive disorder (MDD) and could result in neural degeneration. The interaction of EDCs with hormonal receptors is the best-described mechanism of their biological activity. However, the dysregulation of the hypothalamic-pituitary-gonadal adrenal axis has been reported and linked to neurological disorders. At a worldwide level and in Mexico, the incidence of MDD has recently been increasing. Of note, in Mexico, there are no clinical associations on blood levels of EDCs and the incidence of the MDD. Methodology: Thus, we quantified for the first time the serum levels of parent compounds of two bisphenols and four phthalates in patients with MDD. The levels of di-ethyl-hexyl-phthalate (DEHP), butyl-benzyl-phthalate (BBP), di-n-butyl phthalate (DBP), and di-ethyl-phthalate (DEP), bisphenol A (BPA), and bisphenol S (BPS) in men and women with or without MDD were determined with a gas chromatograph-mass spectrometer. Results/conclusion: We found significant differences between concentrations of BBP between controls and patients with MDD. Interestingly, the serum levels of this compound have a dysmorphic behavior, being much higher in women (~500 ng/mL) than in men (≤10 ng/mL). We did not observe significant changes in the serum concentrations of the other phthalates or bisphenols tested, neither when comparing healthy and sick subjects nor when they were compared by gender. The results point out that BBP has a critical impact on the etiology of MDD disorder in Mexican patients, specifically in women.

## 1. Introduction

According to the World Health Organization (WHO), MDD is a mood disorder with hormonal, neurochemical, and inflammatory alterations [1,2,3,4,5,6]. It has been reported that MDD is present twice as much in females than in males [7]. It is recognized that age, genetic elements, sociocultural factors, and hormonal status can contribute to the sex difference in MDD. Nevertheless, environmental pollutants (industrial compounds, plastics and plasticizers, fungicides, and pesticides) have recently emerged as a crucial factor that must be considered in this disorder [8].

Currently, exposure to different environmental pollutants known as EDCs has been associated with different neurological alterations, including attention deficit/hyperactivity disorder and autism spectrum disorder [9,10]. Certain EDCs such as DEHP can dysregulate the hypothalamic-pituitary-gonadal adrenal axis, which is crucial for reproductive and neuronal processes [11]. In particular, phthalates and bisphenols are ubiquitous environmental pollutants that have been related to the homeostatic imbalance of different systems [12,13,14,15]. Both types of compounds are found in daily-use products [16,17]. Worldwide, their presence has expanded to different sources, including food, air, and water, representing a significant health issue [15]. These compounds are lipophilic, which allows them to be easily absorbed by the skin. In addition, changes in pH or temperature favor their release from the plastic matrix to which they are attached, causing their ingestion through the oral cavity. Once inside the body, they can bind to steroid receptors, which, in turn, allows them to differentially modulate the signaling pathways generally activated by endogenous ligands [18,19]. Of note, exposure to both classes of plasticizers, bisphenols, and phthalates during critical periods can predispose to disturbing human health effects [15]. This could suggest that their mother-infant contact route, intake at the perinatal stage, and adulthood could be associated with the predisposition to develop MDD.

MDD involved alterations in neurotransmitter systems, including noradrenergic, cholinergic, serotonergic, and dopaminergic pathways. In this sense, estrogens such estradiol (E2) increases the effectiveness of the serotoninergic system through the transcriptional modulation of 5-HT transporter (SERT or 5-HTT), tryptophan hydroxylase-2 (TPH-2), and monoamine oxidase A and B (MAO), whereby important antidepressant functions have been attributed to E2 [20,21,22,23,24,25]. In fact, the combination serotonin reuptake inhibitors (SSRIs), such as fluoxetine (FLX) and sertraline with E2 as an adjuvant fashion, have demonstrated promising results for elderly depressed women [26,27].

Of note, the aberrant function of MAOs has a fundamental role in the promotion and maintenance of MDD [28]. Supporting the above, it has been documented that MDD in females generally occurs during periods of hormonal (E2) perturbation [29,30,31]. Until now, there is no existing literature about the modulation of different components of the serotonergic system by the EDCs. However, EDCs interrupt the regular action of hormones, thus, they may affect the function of hormone-sensitive organs, including the brain.

On the other hand, estrogen receptors (ERs) are expressed in different subsets of immune cells. Thus, estrogens can exert both anti- and pro-inflammatory effects, depending on the cell context [32]. In addition, the effects of E2 have also been related to the control of Th1 and Th2 responses [33]. Currently, the prevalence of the Th2 response has been clinically associated with the presence of MDD [34]. The effects of different EDCs on the shift of Th1 and Th2 responses have also been little studied. BPA and phthalates have been associated with a polarization of the Th2 response [35,36,37].

Some research groups have examined the serum levels of E2 in patients with MDD, which are around 85–127 pg/mL and 55 pg/mL in the female and male population, respectively [38,39]. To date, there are many studies about the quantification of phthalates and bisphenols in different biological fluids [40,41,42,43]. It is important to remark there are no reports on their serum levels in patients with MDD worldwide, although various studies support the association between the deleterious effects of EDCs at the neuronal level. According to this, animal studies have exposed the dimorphic damaging role of EDCs at the neuronal level, specifically in the limbic system [44]. In addition, the exposure of different types of EDCs are related with alterations in psychomotor activity and onset of mental disorders [45].

Considering that these substances are found in many sources, and humans are constantly exposed to them, we decided to evaluate the serum concentrations of specific phthalates and bisphenols in patients with MDD in Mexico City. In the case of phthalates, two of them correspond to compounds with high molecular weight, found mainly in plastic devices; and the other two compounds correspond to low molecular weight, which are primarily found in cosmetic and personal use products. Concerning bisphenols, we measured the BPA and one of its primary analogs, BPS.

Serum or urinary levels of different hydroxylated or glucuronide metabolites of phthalates and bisphenols have been evaluated by different research groups [40,41,46]. BPA is rapidly metabolized to an inactive metabolite (glucuronic form) and excreted from the body with a half-life around 5 h [47,48]. Of note, the half-life of phthalates depends on their molecular weight. Low molecular weights (DEP and DBP) are hydrolyzed and quickly converted in monoesters and excreted, meanwhile phthalates with high molecular weight (DEHP and BBP) are also first hydrolyzed and then metabolized in a multistep oxidative pathway [41,49]. However, it has been reported that the enzyme β-glucuronidase is present in various tissues; this enzyme can give the unconjugated form of BPA (active metabolite) and release it into the body [50]. Due to its lipophilic character, BPA can migrate and store in different tissues, bringing the concept of bioaccumulation into the body. Scientific evidence has questioned this notion with different arguments. humans and animals are in constant exposure to BPA and other types of substances. In general, since it has been demonstrated that BPA can be reverted to its active form, then could the unconjugated form of the parental metabolites be measured? Would it be necessary to quantify different pollutants at the blood level and in tissues where they bioaccumulate to have an idea of the possible global damage in the body [47]? Considering these concerns, we decided to evaluate the serum concentrations of parent compounds of phthalates and bisphenols, instead of their metabolites, in patients with MDD.

## 2. Materials and Methods

### 2.1. Patients/Study Population

The outpatient clinic of the Instituto Nacional de Psiquiatria Ramón de Fuente in Mexico City assessed 98 individuals and recruited 14 Mexican patients that met inclusion criteria from January 2015 to December 2018. Patient recruitment was conducted according to the Declaration of Helsinki, and the clinical experimental procedures set out in NC150048SECITI research protocol, approved by the ethics committee of Instituto Nacional de Psiquiatría, México.

The inclusion criteria for this study included participants without medical illnesses, without a history of allergies or allergic reactions. Criteria also included low coffee (2 cups/day), alcohol (3 measures/week), or tobacco (7 cigarettes/day) intake. Women who were pregnant were excluded from the study.

Psychiatrists diagnosed all subjects from Instituto Nacional de Psiquiatría, who applied the validated Spanish version of the Mini-International Neuropsychiatric Interview [51,52,53], a standardized diagnostic interview based on DSM-IV criteria. Clinical status was measured with the 21-item Hamilton Depression Scale (HDRS) and the 21-item Beck Depression Inventory (BDI) [54,55,56]. Blood samples of different patients between 30–65 years with MDD were collected for analysis. Table 1 shows Demographic characteristics of the patients.

### 2.2. Healthy Volunteers

A total of 53 healthy volunteers were matched by age with patients with MDD. Healthy volunteers were recruited from the general population from January 2015 to December 2018. Clinical and laboratory assessments of control subjects fell within typical reference values (data not shown). The MINI confirmed that they did not have any mental disorder and all of them were free of any medication at least 3 weeks before blood and urine sampling. The demographic characteristics of this group are shown in Table 1.

### 2.3. Collection of Serum Samples

For this study, after a detailed explanation of the study aims, all participants signed a written informed consent for experimentation with human samples. This work was conducted in accordance with the Declaration of Helsinki, and the Ethics Committee approved the protocol of Instituto Nacional de Psiquiatria Ramón de la Fuente Muñiz.

### 2.4. Sample Treatment

Serum samples healthy individuals and patients with depression were centrifugated to obtain the serum. After that, an organic methanol-based extraction protocol was performed, obtaining a dry lyophilized extract [57]. The extracts were reconstituted and derivatized by adding 50 µL of N-Methyl-N-trimethylsilyl-trifluoroacetamide (MSTFA) and heating at 80 °C for 30 min in a dry block heater. After derivatization, 40 µL was taken and transferred into a 200 µL vial insert. A total of 10 µL of deuterated dicyclohexyl phthalate (2.5 ng/µL) was added as internal standards immediately prior the injection into a gas chromatograph coupled to a mass spectrometer (GC-MS).

### 2.5. Data Analysis

Chromatographic analyses were carried out with Enhanced Data Analysis software (Agilent Technologies, Santa Clara, CA, USA). The peak identity was analyzed comparing the retention time and analyte standards, which was confirmed by mass spectrum. Internal standard calibration curves were also employed for quantitation.

### 2.6. Reagents and Chemicals

Phthalate diester standards diethyl phthalate (DEP), di-n-butyl phthalate (DBP), butyl-benzyl phthalate (BBP), and bis-ethyl-hexyl phthalate (DEHP) were purchased from Chemservice Inc. (West Chester, PA, USA), Deuterated dicyclo-hexyl phthalate was from Accustandard (New Haven, CT, USA). Bisphenol-A, Bisphenol-S, MSTFA were acquired from Sigma-Aldrich (St. Louis, MO, USA). Methanol and ether were purchased from MilliporeSigma (Burlington, MA, USA).

### 2.7. GC-MS Conditions

Gas chromatograph-mass spectrometer analyses were performed (7890-B/5977-B), Agilent Technologies, Santa Clara, CA, USA) with a quadrupole mass filter. Each sample was analyzed in duplicate. A 60 m DB-35ms capillary column (250 µm × 0.25 µm) was used for chromatographic separation. High-purity helium was used as the carrier gas at a flow rate of 1.2 mL/min. The initial oven temperature was set to 80 °C for 1 min and then increased by 20 °C/min to 320 °C, with a 7 min hold. The mass spectrometer was operated in electronic ionization mode (70 eV) in scan mode (25–430 Da). The temperatures were 300 °C for the transfer line, 230 °C for the ion source, and 150 °C for the quadrupole. Of note, the GC-MS conditions correspond to a preliminary optimization. In addition, for the analysis of each compound, the retention time of peaks was calculated and compared with a mass spectra library, as was previously described in [58]. Table 2 shows the quantitative analysis parameters used for GC-MS analysis.

### 2.8. Statistical Analysis

The statistical differences about the concentration of different compounds among the groups were determined by a non-parametric analysis, using the Mann–Whitney U test for paired comparisons. For the analysis, the specialized software package GraphPad Prism 6 version (San Diego, CA, USA) was used. *p* < 0.1 was considered statistically significant. The sample size was 54 subjects for the control group and 14 subjects for the case (patients with MDD) group. The type of study corresponded to a cross-sectional study, comparing the levels of EDCs in patients with MDD and healthy individuals, as well as a comparison of the levels of EDCs between men and women. Of note, we considered for the statistical tests a confidence interval of 90% and a two-tailed test.

## 3. Results

### 3.1. Serum Levels of Environmental Pollutants in Healthy Individuals and Patients with MDD

**Figure 1:**
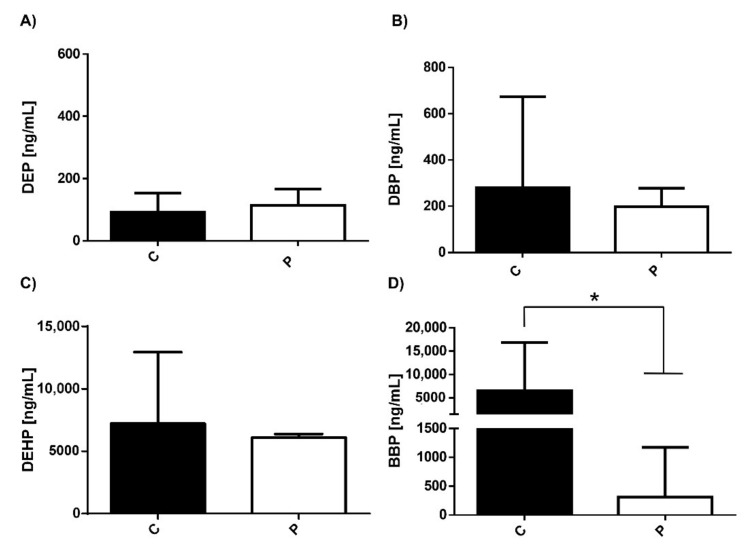
Levels of phthalates in controls and patients with depression. Serum concentration levels of phthalates (**A**) DEP, (**B**) DBP, (**C**) DEHP, and (**D**) BBP were measured in healthy individuals (C) (53 subjects/black bar) and in patients with MDD (P) (14 subjects/white bar). Bars represent the mean ± S.D. * *p* < 0.1 was considered statistically significant. In the case of the comparison between C and P groups we found a Mann–Whitney U value of 191.5.

We analyzed the serum concentrations of different phthalates in the control group (C) and patients with depression (P), (Figure 1). The results showed that both controls and patients have similar basal levels (~200 ng/mL) of all phthalates tested. However, significant differences between the controls and patients were found in BBP levels (*p* = 0.0019). It should be noted that in patients with depression there were 10 times lower ranges (<500 ng/mL) of BBP compared to healthy individuals (~5000 ng/mL) (Figure 1D).

**Figure 2:**
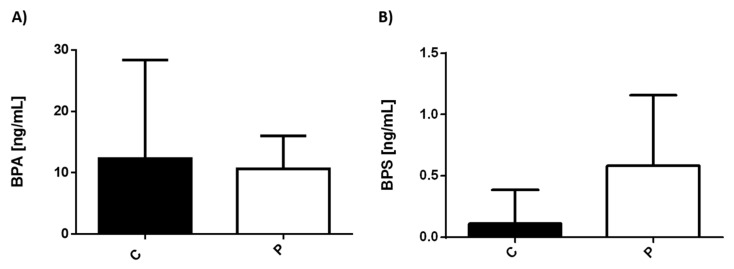
Levels of bisphenols in controls and patients with depression. Serum concentration levels of (**A**) BPA, (**B**) BPS were measured in healthy individuals (C) (53 subjects/black bar) and in patients with MDD (P) (14 subjects/white bar).

We also analyzed BPA and BPS serum levels in the two experimental groups. (Figure 2). BPS levels were low in both controls and sick individuals. However, there was a significant difference between them, showing higher levels in patients with depression than that in the healthy counterpart (Figure 2B). The levels of BPA were not significantly different between the two experimental conditions.

### 3.2. Serum Levels of Environmental Pollutants in Patients with MDD Separated by Gender

**Figure 3:**
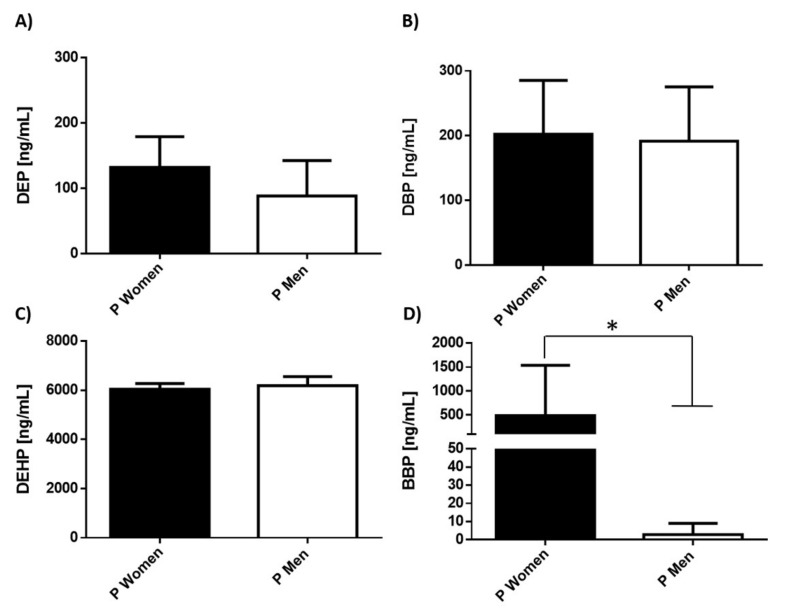
Levels of phthalates in male and female patients with depression. Serum concentration levels of phthalates (**A**) DEP, (**B**) DBP, (**C**) DEHP, (**D**) BBP were measured in women (9 subjects/black bar) and men (5 subjects/white bar). Bars represent the mean ± S.D. * *p* < 0.1 was considered statistically significant. In the case of the comparison between C and P groups we found a Mann–Whitney U value of 9.0.

Because the incidence of depression has a dimorphic tendency, we consider it important to compare the levels of pollutants in male and female patients with depression. The results showed that DEP, DPB, and DEHP serum levels were similar in both genders (Figure 3). However, outstandingly, the levels of BBP were significantly different in women with depression (*p* = 0.0584), showing a more significant increase (>50-fold) compared with men (Figure 3D).

**Figure 4:**
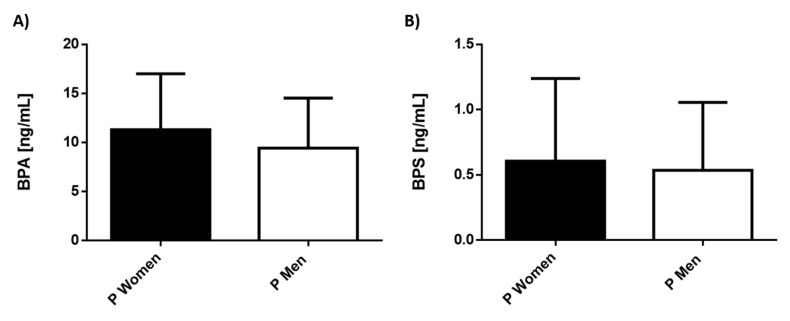
Levels of bisphenols in male and female patients with depression. Serum concentration levels of phthalates (**A**) BPA, (**B**) BPS were measured in women (9 subjects/black bar) and men (5 subjects/white bar). Bars represent the mean ± S.D.

There were no dimorphic differences in bisphenols, and the serum values found were similar in men and women, Figure 4.

## 4. Discussion

This study determined the serum concentration of parent compounds of different phthalates and bisphenols in healthy individuals and patients with MDD.

MDD is a mood disorder of multifactorial origin, which is considered a global health emergency due to the number of patients worldwide and the costs they cause to public health in all countries. Research on this condition has focused on aspects of genetic susceptibility, hormonal, neurochemical, and inflammatory alterations [1,2]. MDD is characterized by the decrease in the production of biogenic amines and globally is accompanied by the dysfunction of the neuro-immuno-endocrine systems. In this regard, estradiol (E2) is widely known to play an important role in the regulation of critical components of the optimal function of the serotonergic system and enzymes that degrade monoamines [2]. Despite the enormous effort in pharmacological research for MDD developed since the last century, there are still no effective pharmacological treatments and therapeutic adherence is low, which increases therapeutic failure. All above contributes to having a high number of patients with this condition.

One point to consider is that, despite reports on the possible participation of environmental pollutants such as BPA, DBP, BBP, and DEHP in the establishment and aggravation of MDD, few studies evaluate their levels in properly diagnosed patients and explore the mechanisms associated with the development of the symptoms of these conditions. Considering that various EDCs such as phthalates and bisphenols mimic hormone actions, we decided to evaluate the concentrations of various phthalates and two bisphenols in the serum of patients with depression and compare their levels with healthy ones, as well as between male and female individuals. It should be noted that the compounds we choose are widely used in commonly used products.

We mainly found significant differences between concentrations of BBP between controls and patients with MDD. Interestingly, the serum levels of this compound have a dimorphic behavior, being much higher in women than in men. BBP is a high-molecular-weight phthalate with an asymmetrical structure, used as a plasticizer in PVC products [59]. BBP has demonstrated an etiological association with endometriosis that directly correlates with high blood levels of this contaminant [60]. Supporting the above, BBP can modulate different hormone-dependent genes and it has been demonstrated to be the most active estrogenic phthalate among others [61,62]. Of note, different phthalates have been shown to decrease serum levels of E2 with a concomitant prolonged estrous cycle, causing anovulation in rats. This effect was due to the suppression of aromatase gene expression, the responsible enzyme of E2 synthesis [63]. Considering that the optimal levels of E2 are implicated in the regulation of multiple systems and have an essential antidepressant function, it will be important to determine levels of contaminants (EDCs) and E2 in patients with MDD simultaneously, to be able to replace the basal levels of this hormone. In addition, exposure to different phthalates has harmful pleiotropic actions [64], which is still unknown at the brain level.

The serum levels of different metabolites of BBP have been determined, in women (~140 ng/mL) and in men (85–100 ng/mL) [65,66]. We observed a 35 times higher concentration of this compound at the serum level in the female population. However, it is important to remark that we measured the parent compound instead of some metabolites. Our results can be supported in previous reports where women have greater exposure to phthalates than males of the same age, possibly due to increased use of cosmetics and medication, considering that phthalates are employed in the manufacture of capsules or the drug packaging [67,68]. A recent study by Shao-hui Zhang 2018 et al., evaluated the distribution of different phthalates (parent compounds, not their metabolites) in the serum of patients with high blood pressure in China [69]. Interestingly, they found deficient concentrations of BBP (~0.5 µg/L) in the population included. However, it is important to highlight that of 454 participants, 336 were men, and 138 were women [69], supporting our results.

On the other hand, an explanation of why BBP can induce MDD is based on its lipophilic characteristics [70], which could favor its migration and storage at the central nervous system level. In addition, BBP exposure also causes a decrease in serotonin (5-HT) levels [71], which then, in turn, attends to the activation of adenylyl cyclase by G protein-coupled receptors (GPCRs). This enzyme catalyzes the formation of cAMP from ATP in an energy-dependent manner. Thus, the activating of protein kinase A can be impaired, which decreases the levels of CREB phosphorylation. Concomitantly, the decrease of pCREB attenuates the effects of CREB downstream. Thus, oxidative damage and pathological alterations in mice brains and the impaired behavioral performance were evidence [71].

Regarding the cellular effects of other phthalates, it is known that DBP increases the concentration of reactive oxygen intermediates [72], which might also cause significant damage in neurons. There are also reports about different phthalates highlighting the alterations that phthalates evoked in sex hormones and different enzymes involved in their biosynthesis or catabolism in male and female animal models [73]. The disturbances in different hormone levels found in in vivo models might favor a physiological condition comparable with MDD disease in humans, such as cognitive decline, impaired learning, and memory. Supporting these facts, perinatal phthalate exposure seems to induce hippocampal impairment involving downregulation of androgen and estrogen receptor expression in mice [74], effects caused by the crossing of phthalates through the placental barrier [75,76], which suggests that phthalates also cross the blood–brain barrier. Moreover, exposure to phthalates and other EDCs in early periods of life can affect neural function due to the impairment of methylation profile in the brain [45], conferring greater susceptibility to neuronal disorders such as MDD into adulthood. It has also been described that several EDCs are involved in epigenetic modulation and may even promote the expression of different miRNAs [77]. Both processes have been associated with the development of depression [78]. More in-depth studies on the relationship between EDCs and miRNAs modulation in the promotion of depression deserve future research.

On the other hand, not many studies have evaluated the dimorphic role of phthalates in the brain and their functions in rats or mice; however, there is evidence that the postanal exposure of DHEP in rats may harm the development of the hippocampus in males but not in females [79]. The explanatory mechanism by which DEHP caused these effects was that in the female rat hippocampus, DEHP alters the lipid profile, leading to elevated levels of phosphatidylcholine and sphingomyelin. In contrast, the effect of DEHP was absent in the hippocampus of male rats. Thus, the authors postulated that the upregulation of hippocampal lipids could have a neuroprotective role in DEHP-exposed female rats [80].

In the case of BPA, it has been observed that the offspring of rats exposed to BPA have dimorphic repercussions on the nervous system, such as higher activity, a lower avoidance memory, and larger locus coeruleus than the male controls, while the BPA-exposed group did not show any sexual dimorphism. From what can be concluded, the accepted no-observed-adverse-effect level (NOAEL; 50 mg/kg per day) may not be the best reference to see damage at the brain level [81]. However, we cannot make further statements due to the small male population included in this study.

Regarding bisphenols, our results denoted that the levels of BPA and BPS in the individuals included were between 10 ng/mL and 0.5 ng/mL, respectively. They are close with the data reported by other authors in healthy individuals [82,83,84]. However, we did not find significant differences between the levels of phthalates neither in healthy populations nor in patients with MDD. This may be due to the limited number of patients with this disease enrolled in this study. Recently, more biological evidence has linked the role of environmental exposure and the generation of disorders such as depression, although there is still a lack of clinical data that strongly supports this relationship [85]. We found only one study correlating BPA exposure with some mental disorders, such as depression. Braun et al. enrolled 3-year-old children of 244 mothers; the authors found higher gestational BPA concentrations associated with higher anxiety, hyperactivity, and depression, especially in girls [86].

Currently, there are research groups that, with the help of bioinformatic tools, focus on clarifying the effects of EDCs in the development of brain diseases and mental disorders. In this sense, bisphenols and phthalates generate interaction networks of molecules involved with different processes such as neuron growth and survival, neuroplasticity, synapses, and cognitive function. These alterations have made known in a comprehensive way the role of EDCs in Alzheimer’s Disease (AD), Parkinson’s Disease, autism spectrum disorder, and even brain neoplasms [87]. So far, there are no similar studies of the relationship of these compounds with the development of MDD. Still, the same approaches could be generated to elucidate the effects of these compounds on MDD.

It is important to remark that we carried out a deep search in different databases, including PubMed with the terms EDCs, MDD, comparison of mental disorders in women and men due to EDCs, and we did not find reports dating back to the year 2000, nor between the years 2000–2021.

We consider that the present study offers clinical knowledge that strongly suggests that EDCs, particularly BBP, play an important role in the generation/promotion of psychiatric illnesses such as MDD. However, we are aware that this work has several limitations: (1) we cannot compare the serum concentrations of EDCs with other works, as we measured the parent compounds and not the metabolites, although the data obtained are not very different from what was reported; (2) it will be essential to include more patients with MDD to make stronger assumptions; and finally (3) the serum measure of E2, other hormones, neurotransmitters, lipid and methylation profile, or different cytokines will be important to provide a global landscape about the effect of different EDCs and MDD.

## 5. Conclusions

Our results suggested that phthalate exposure, particularly BBP, might increase MDD in female adults. However, our findings warrant further studies in larger population. This study lays the foundations for making associations between exposure to EDCs and the development of MDD since there is insufficient evidence on this aspect.

Additionally, there are several limitations for understanding the effects of EDCs in humans due to the set of pollutants to which we are exposed and the extended exposures during the early lives to adult outcomes, and the molecular mechanisms that explain dimorphic differences in their effects.

## Figures and Tables

**Table 1:** Demographic characteristic of study sample.

	Patients *n* = 14	Healthy Volunteers *n* = 53
Age (years)	34.1 ± 9.1	34.1 ± 9.1
Sex (male/female)	5/9	7/46
BMI (kg/m^2^)	24.8 ± 2.5	24.8 ± 2.5
Education (years)	10.6 ± 4.93	14 ± 5.6
Family history (yes/no)	5/9	NA
First episode	6	NA
Recurrent episode	8	NA

**Table 2:** Quantitative analysis parameters.

Compound	Linear Range (pg)	Slope	Intercept	r^2^	Monitored Ions
BPA-TMS	0.1–10	0.0078	0.0375	0.9902	357, 358, 372
BPS-TMS	0.1–10	0.0025	0.004	0.9856	394, 379
DEP	10–10,000	0.002	0.2031	0.9948	149, 177, 76
DBP	10–10,000	0.0045	0.4721	0.9998	149, 205, 223
BBP	10–10,000	0.0019	0.0548	0.9999	149, 91, 206
DEHP	10–10,000	0.0027	0.2162	0.9995	149, 176, 279

## Data Availability

Data availability can be requested by writing to the corresponding author of this publication.
